# Anti-hyperlipidemic and anti-atherosclerotic effects of *Pinus eldarica* Medw. nut in hypercholesterolemic rabbits

**DOI:** 10.1186/s40199-015-0114-9

**Published:** 2015-06-09

**Authors:** Hasan Fallah Huseini, Maryam Sotoudeh Anvari, Yaser Tajallizadeh khoob, Shahram Rabbani, Farshad Sharifi, Seyed Masoud Arzaghi, Hossein Fakhrzadeh

**Affiliations:** Medicinal Plants Research Center, Institute of Medicinal Plants, ACECR, Karaj, Iran; Clinical and Surgical Pathology Department Tehran Heart Center, Tehran University of Medical Sciences, Tehran, Iran; Endocrinology and Metabolism Research Center, Endocrinology and Metabolism Clinical Sciences Institute, Tehran University of Medical Sciences, Tehran, Iran; Experimental Research Center, Tehran Heart Center, Tehran University of Medical Sciences, Tehran, Iran; Elderly Health Research Center, Endocrinology and Metabolism Population Sciences Institute, Tehran University of Medical Sciences, 4th floor, No 4, Ostad Nejatollahi Street, Engelab Avenue, Tehran, Iran

**Keywords:** *Pinus eldarica* nut, Rabbits, Atherosclerosis, Hypercholesterolemia

## Abstract

**Background:**

Previous studies suggest that chemical constituents present in *Pinus eldarica* Medw (*P. eldarica*) nut possess antioxidant properties that may positively influence lipid profile.

The present study was conducted to evaluate the efficacy of *P. eldarica* nut on the experimental atherosclerosis development in hypercholesterolemic rabbits.

**Methods:**

Forty male 6 months old white New Zealand rabbits (1.8–2 kg) were randomly assigned into five equal groups. One group was kept as control (normal) group, fed on standard rabbit diet and other 4 groups were fed on high cholesterol diet (HCD). Out of four HCD groups one group was kept as control (HCD) and other three groups were treated with different doses (50, 100 and 200 mg/kg/day) of *P. eldarica* nut for 8 weeks. Percentage of aortic wall area changes as indication of atherosclerosis development and fasting blood cholesterol, LDL, HDL and triglyceride levels were determined in all groups.

**Results:**

The results indicate that fasting blood cholesterol and aortic atherosclerotic involvements in 200 mg/kg/day and 100 mg/kg/day *P. eldarica* nut extract treated groups significantly decreased as compared to the high cholesterol-diet control group.

**Conclusion:**

*P. eldarica* nut lowers blood cholesterol level and aortic atherosclerotic involvement in hypercholesterolemic rabbits.

## Introduction

Atherosclerotic vascular disease is a major cause of morbidity and mortality worldwide [1]. Dyslipidemia is a crucial risk factor for atherosclerosis and oxidative stress following free radical-mediated oxidation of low-density lipoproteins is associated with progression of atherogenesis and its vascular complications [2–4]. In the search for plant foods that provide cardiovascular benefits, nuts have recently enticed attention. Among nuts, the Iranian pine, *Pinus eldarica* Medw (*P. eldarica*) nuts are used as food. *P. eldarica* belongs to the botanical family pinaceae and naturally grows in the Transcaucasian region between Europe and Asia, and it is also spread in Iran, Afghanistan and Pakistan [5, 6].

In Russia and the Central Asian countries the *P. eldarica* needles, buds, resin and nuts have been widely used in traditional medicine for the treatment of bronchial asthma, and various skin diseases [7, 8]. Several components such as β-caryophyllene, α-pinene, longifolene, α-humulene, δ-3-carene and β-pinene with antioxidant properties have been reported in the *P. eldarica* nut oil [9].

In our previous study high concentrations of total polyphenols and fatty acids have been detected in *P. eldarica* nut as indication of its antioxidant properties [10]. Experimental studies strongly support the efficacy of polyphenols and unsaturated fatty acids in the treatment of chronic diseases including cardiovascular disorders [11–14]. The present study was conducted to evaluate the possible anti-hypercholesterolemic and anti-atherosclerotic effects of *P. eldarica* nut in hypercholesterolemic rabbits.

## Methods

### Chemicals

Methanol, phosphoric acid, chloroform, acetonitril and sodium chloride were purchased from Merck Company (India) nn. Ethyl acetate was purchased from Sinopharm Chemical Reagent Company (China). Polyphenol standards: (+) - catechin, (−) - epicatechin, Gallic acid, vanillic acid, *para* coumaric acid, ferullic acid*, ortho* coumaric acid and tyrosol were purchased from Sigma-Aldrich Corporation (Germany). Cholesterol powder was purchased from Solvay Duphar Co., Belgium.

### Plant material

*P. eldarica* cones were collected from Chitgar Forest Park (West of Tehran). The cones were collected between June and July of 2010. The plant was identified by M. Ahvazi and herbarium specimen was preserved in the herbarium of Medicinal Plants Institute (ACECR) with Herbarium code of 689. *P. eldarica* cones were dried in a dark place at room temperature. The nuts were removed from cones and grounded to powder by grinder.

### Plant extract

The hydroalcholic extract of *P. eldarica* nut powder was prepared using 70 % ethanol in water, using percolation method at room temperature. The powdered plant material was soaked initially in a solvent in a percolator and then sufficient amount of the solvent was added to cover material and kept for 24 h with occasional stirring. Then the outlet of the percolator was opened and the liquid contained therein was allowed to drip slowly. The procedure was repeated twice and the combined extractions were clarified by filtration and concentrated to dryness on rotary evaporator at a maximum of 40 °C temperature and under reduced pressure.

### Polyphenol determination

The polyphenol content of *P. eldarica* nut was determined by the High-performance liquid chromatography (HPLC) method developed by Dogan et al. [15]. In brief a standard solution was prepared by dissolving 10 mg of (+) catechin (sigma), epicatechin (sigma), and other phenolic compounds in 10 ml methanol and diluting this solution with methanol HPLC grade. The range of concentration was between 50 and 1000 μg/ml and standard curve was linear (R^2^ ≥ 0.998). Then 20 μL of sample solution was injected to the HPLC and the chromatogram was recorded at 280 nm. First, 20 μl of standard solution was injected and the chromatogram was recorded at 280 nm. Next, we compared the area of the peaks in standard and sample chromatograms and calculated the amount of polyphenols in *P. eldarica*. Total phenol was computed from measurement of individually detected phenols [16].

### Animals and diet

Forty male 6-month-old white New Zealand rabbits (1.8–2 kg) were purchased from Razi Research Institute, Karaj, Iran. High cholesterol diet (HCD) was prepared by adding of 1 g cholesterol powder and 3 g corn oil to 96 g of standard laboratory food.

### General procedures

Rabbits were housed individually in cages in temperature-controlled room (24 °C) under a 12-h light/dark cycle with free access to food and water in the Animal Research Center of Institute of Medicinal Plants. The experimental protocol was approved by the Iranian Institutional Animal Ethics Committee and was conducted according to the Iranian Institutional Animal Care Guidelines for the use and care of experimental animal, drug, dose, and treatment schedule.

After a 2-week adaptation period, rabbits were randomly assigned to five equal groups: control (normal) group fed on standard a diet, HCD control group fed on HCD (1 % cholesterol), and three groups fed on HCD and treated with *P. eldarica* nut in three different doses (50, 100 and 200 mg/kg/day). The extract in the dosages of 50, 100, 200 mg was mixed with little amount of rabbit pellets and fed orally one hour before feeding. The whole experiment lasted 8 weeks. Upon termination of the study biochemical analysis of serum lipids and pathological evaluation of aortas were performed.

### Biochemical analysis

At the end of the study, the overnight fasting blood sample was taken from marginal ear veins for lipid analysis. Fasting blood cholesterol (Cho) LDL, HDL and triglyceride (TG) levels in sera were measured using enzyme assay kits (Pars Azmun Co., Iran).

### Pathological analysis

At the end of the study, rabbits were killed by chloroform (overdose) and their aortas were separated up to diaphragm. The aortas were then divided into the proximal, middle, and distal segments. The aortic specimens were dehydrated in a graded series of alcohol xylene and embedded in paraffin for light microscopic examination. From each paraffin block, four sections of 4 mm^2^ were cut and stained with hematoxylin and eosin. All sections were evaluated microscopically for fatty streak, foam cells, extracellular lipid core, as atheromatous plaque elements. The degree of vascular injury and atherosclerosis was quantitatively measured based on lesion area (on scale of mm^2^) with use of a color image analyzer (E200; Nikon, Tokyo, Japan) [17]. Both the surgeon and the pathologist were blinded to the control and experiment groups.

### Statistical analysis

All values are expressed as means ± SE. Data were analyzed by one way ANOVA, and all differences were inspected by Duncan’s multiple test. Differences were considered to be significant at *p <* 0.05.

## Results

The polyphenol content of the *P. eldarica* nut is presented in Table 1. Considering this method more than 8 polyphenols were identified in *P. eldarica* nut in which catechin, epicatechin and tyrosol were highest in concentration.

**Table 1:** Percent of polyphenols in *P. eldarica* nut mean ± SD [16]

Polyphenols	Percent
Catechin	10.1 ± 0.18
Epicatechin	10.3 ± 0.18
Gallic acid	1.6 ± 0.09
P. coumaric acid	1.4 ± 0.12
Ferullic acid	1.7 ± 0.21
O. coumaric acid	0.12 ± 0.02
Tyrosol	29.1 ± 0.08
Dimers of catechin and epicatechin	7.5 ± 1.06
Unknown	38.18 ± 0.28
Total phenols (ppm)	483 ± 27

**Fig. 1:**
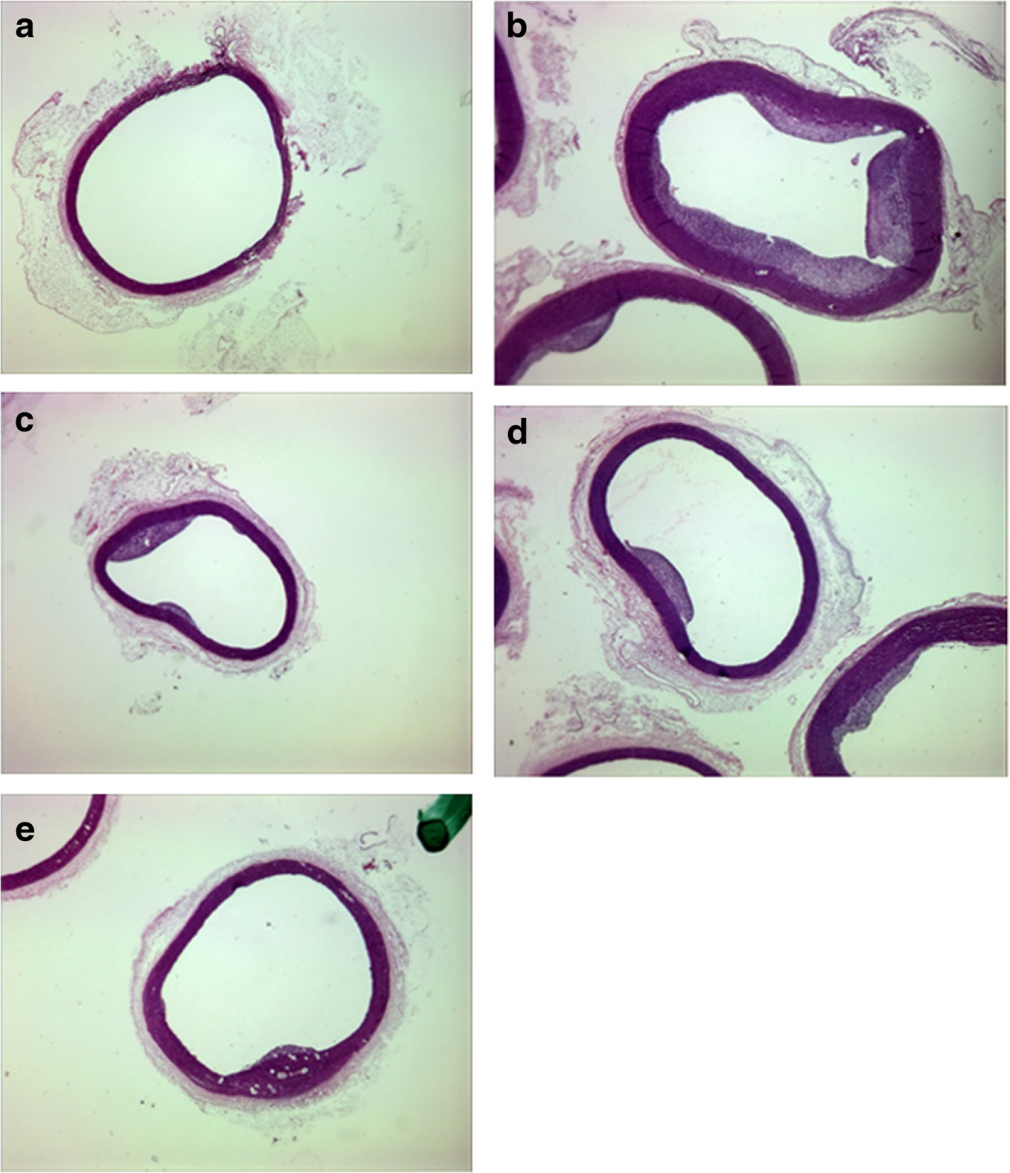
Morphological changes of the aortic wall atherosclerotic involvement. **a** Normal rabbits fed on standard laboratory diet, **b** Rabbits fed on HCD; **c** Rabbits fed on HCD and treated with 50 mg/kg of *P. eldarica*; **d** Rabbits fed on HCD and treated with 100 mg/kg of *P. eldarica*; **e** Rabbits fed on HCD and treated with 200 mg/kg of *P. eldarica*

Results show that feeding the rabbits for 8 weeks with HCD leads to a significant increase in their blood serum total cholesterol, LDL-C and to a lesser extent in TG levels. Results indicate that serum total cholesterol and LDL-C levels significantly decrease in the groups treated with 200 mg/kg/day and 100 mg/kg/day of *P. eldarica* compared to the control group; although it is of note that all of the aortic samples from HCD-fed rabbits showed some degree of atherogenesis and none of them were normal. There were no significant changes in fasting blood TG and HDL-C levels in *P. eldarica* treated groups compared to control group (Table 2) (Fig. 1).

**Table 2:** The fasting blood parameters and aortic wall area percent (mm^2^) after 8 weeks of high cholesterol diet (HCD) fed rabbits treated with *P. eldarica* nut extract at dosages of 50, 100, 200 mg/kg compared with high control HCD fed rabbits (mean ± SD)

Groups	Triglyceride (mg/dl)	Cholesterol (mg/dl)	LDL (mg/dl)	HDL (mg/dl)	Aortic wall area percent (mm^2^)	Percent of aortic wall area changes
Control (normal)	60.4 ± 15.8	31.4 ± 4.5	9.2 ± 4.7	13.2 ± 3.2	0.247 ± 0.032	
Control (HCD)	91.2 ± 48.9	1545.6 ± 510.6	1060.6 ± 360.5	113.4 ± 55.8	0.296 ± 0.049	16.55↑ Compared with control (normal)
	*P* = 0.218*	*P* = 0.003*	*P* = 0.016*	*P* = 0.003*	*P* = 0.005*	
*P. eldarica* nut extract 50 mg/kg	93.6 ± 31.2	1253.0 ± 578.0	834.4 ± 201.2	87.6 ± 17.7	0.276 ± 0.048	6.76↓ compared with control (HCD)
	*P* = 0.929**	*P* = 0.421**	*P* = 0.544**	*P* = 0.351**	*P* = 0.350**	
*P. eldarica* nut extract 100 mg/kg	102.2 ± 37.7	834.4 ± 201.2	552.4 ± 158.2	90.2 ± 15.9	0.261 ± 0.038	11.83↓ compared with control (HCD)
	*P* = 0.700**	*P* = 0.020**	*P* = 0.034**	*P* = 0.410**	*P* = 0.048**	
*P. eldarica* nut extract 200 mg/kg	108.8 ± 28.0	710.4 ± 448.0	438.4 ± 292.2	99.4 ± 11.8	0.259 ± 0.042	12.50↓compared with control (HCD)
	*P* = 0.505**	*P* = 0.025**	*P* = 0.020**	*P* = 0.47**0	*P* = 0.032**	

*P* < 0.05 was considered as statistically significant

*compared with control (normal) group

**compared with control (HCD) group

Morphological changes of the aortic wall atherosclerotic involvement are shown in Fig. 1. The aortic wall area percent in groups treated with 200 mg/kg/day and 100 mg/kg/day *P. eldarica* nut decreased significantly compared to the control group (Table 2).

## Discussion

The results suggest that *P. eldarica* nut extract reduced total cholesterol, LDL-C and extent of aortic atherosclerotic involvement in hypercholesterolemic rabbits. The mechanism underlying inhibition of aortic atherosclerotic progression by *P. eldarica* nut extract may not be solely due to its lipid lowering effect, as the levels of serum total cholesterol and LDL-C were remarkably higher in cholesterol-fed rabbits than in normal ones. Other mechanisms may also play a role for such effect. Increases in serum cholesterol and LDL-C and consequent oxidation of LDL-C are essential steps for development of atherosclerotic plaques [2]. In fact formation of oxidatively-modified LDL-C is the first step of atherogenesis in so-called “oxidation hypothesis” [17]. Moreover it has been shown that susceptibility of LDL-C to oxidation independently correlates with the extent of atherosclerosis [18]. It is now established that inhibition of oxidative stress and lipid oxidation could have beneficial effects on regression of atherogenesis [19]. *P. eldarica* nuts contain high concentrations of polyphenols and flavonoids (Table 1) [10]. Several data suggested that, flavonoids improve dyslipidemia, inhibit low-density lipoprotein cholesterol oxidation and protect vascular endothelium against oxidative damage [20–22]. Furthermore, catechin and epicatechin which are found in high concentrations in *P. eldarica* nuts are also main chemical constituent of other Pinus species barks such as French matitime pine bark extract (pycnogenol) [23]. Several studies have shown the beneficial effects of pycnogenol for health to be mainly due to its antioxidant properties and consequent excellent free radical scavenging function [24–26]. In addition other components such as tyrosol with antioxidant properties which are present in high concentrations in *P. eldarica* nuts may directly or indirectly influence lipoprotein and cellular metabolism against atherogenesis [10, 27].

On the other hand, the observed anti-atherogenic effects of *P. eldarica* nut in the rabbits may also be in part due to appreciable amounts of essential oils such as α-pinene, β-pinene and β-caryophyllene present in this nut [9]. The antioxidant properties of α-pinene, β-pinene and β-caryophyllene are reported in other studies [28]. Of note the limitations of this study were lack of determination of the essential oil components and antioxidant properties of *P. eldarica* nut extract. To our knowledge this is the first trial on the anti-atherosclerotic and hypolipidemic effects of *P. eldarica* extracts on hypercholesterolemic rabbits. In a similar study we also have recently shown the beneficial effects of *Pinus eldarica* nut extract on blood glucose and cholesterol levels in hypercholesterolemic alloxan-induced diabetic rats [29]. As *P. eldarica* nuts are usually safe and used as food, further investigation of their clinical efficacy in the treatment of hypercholesterolemia is suggested.
